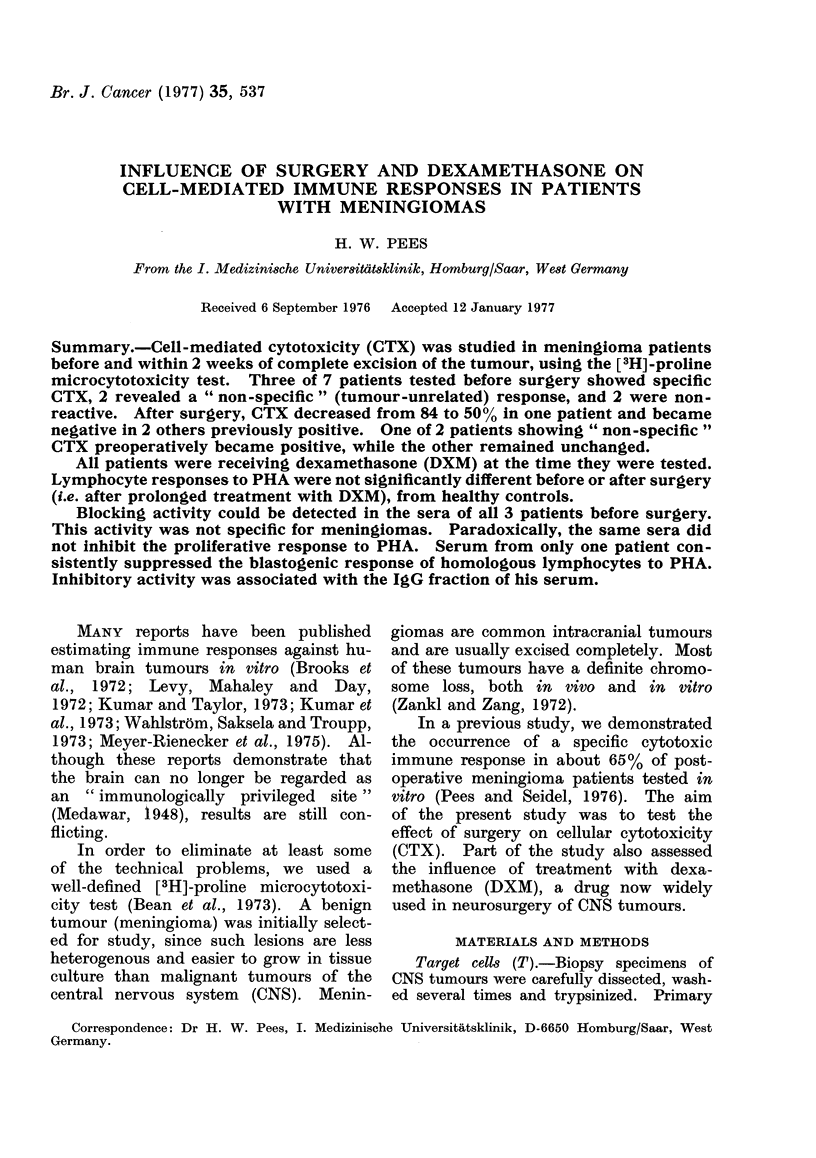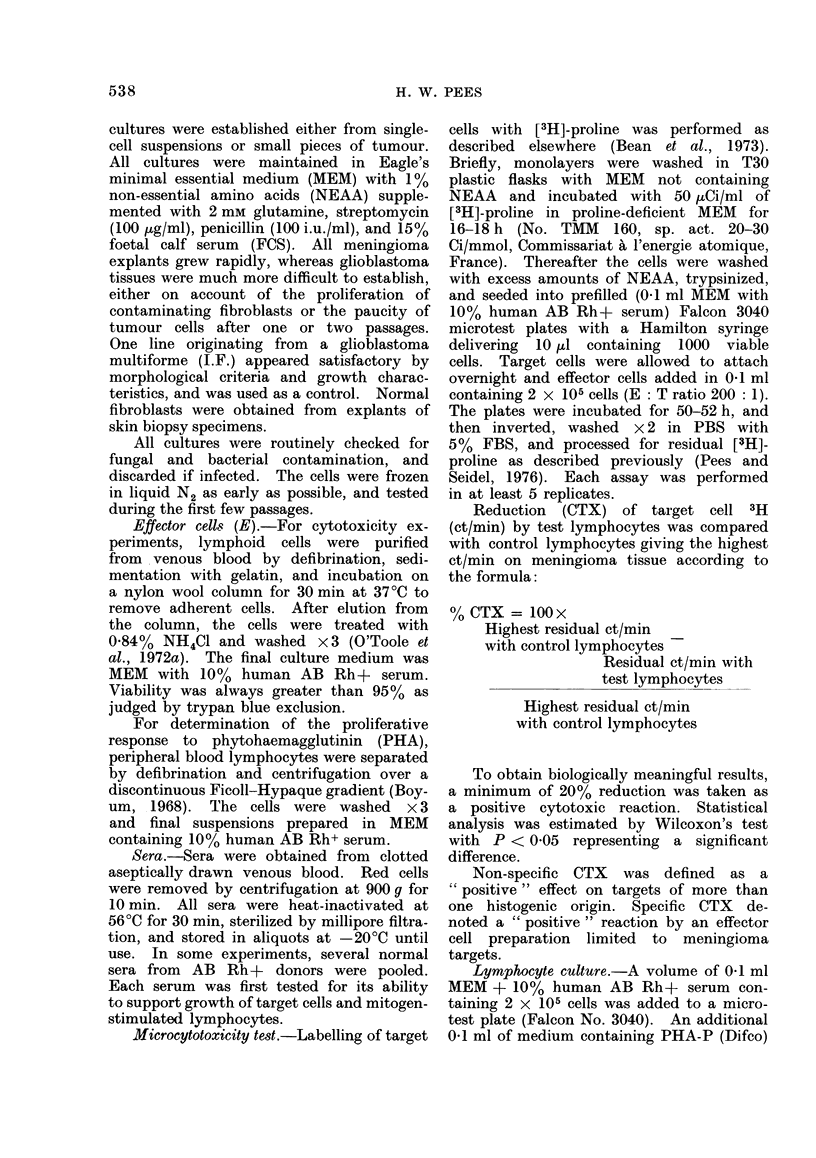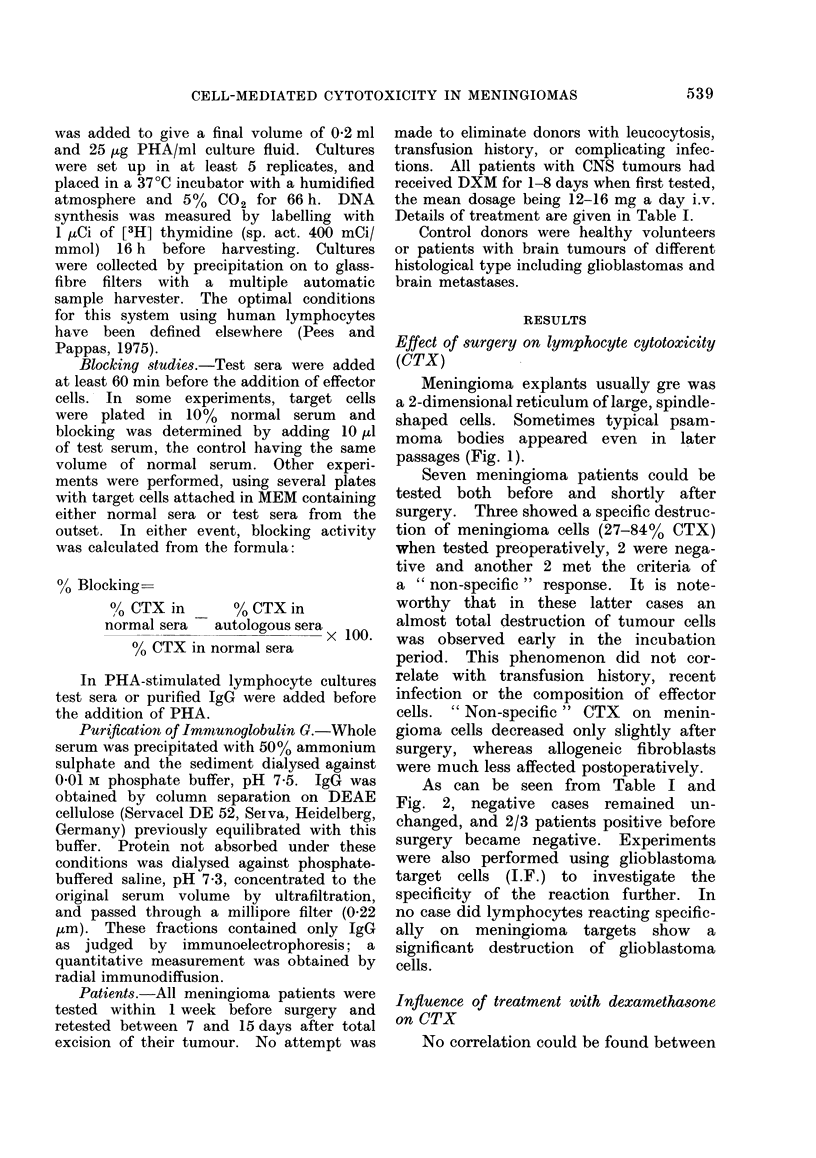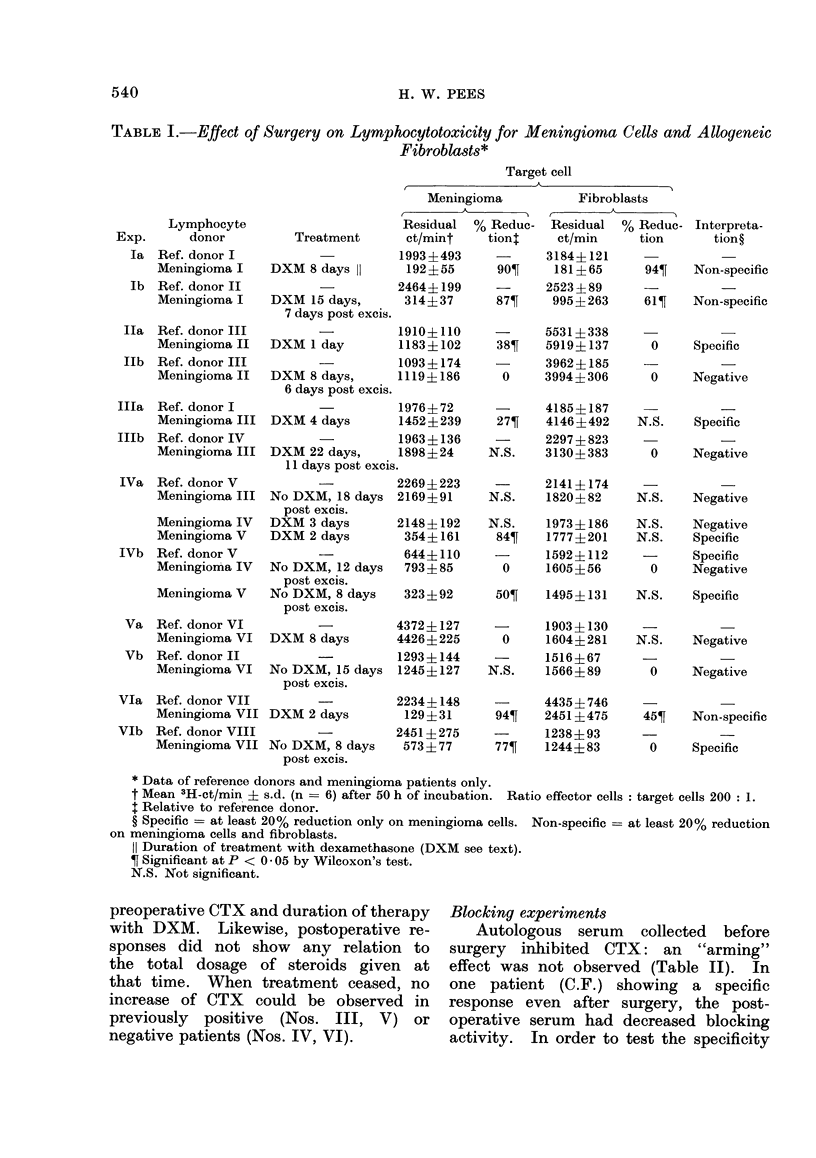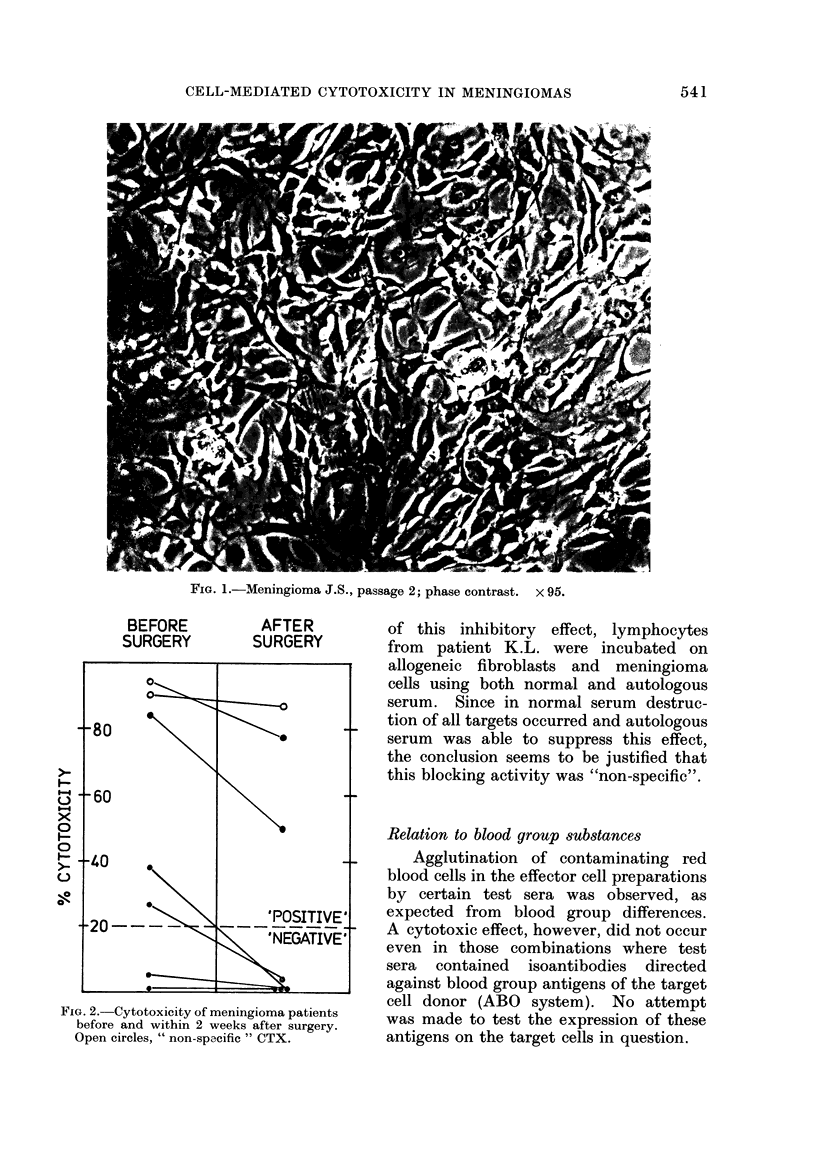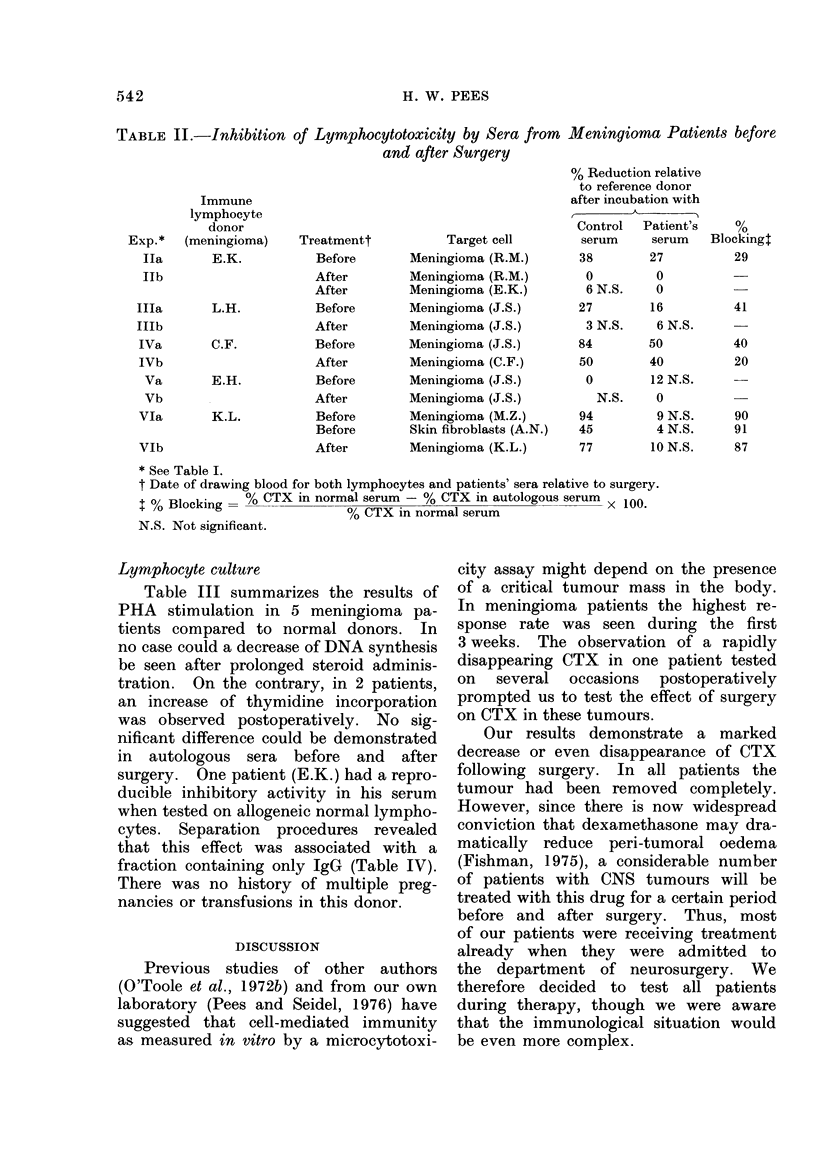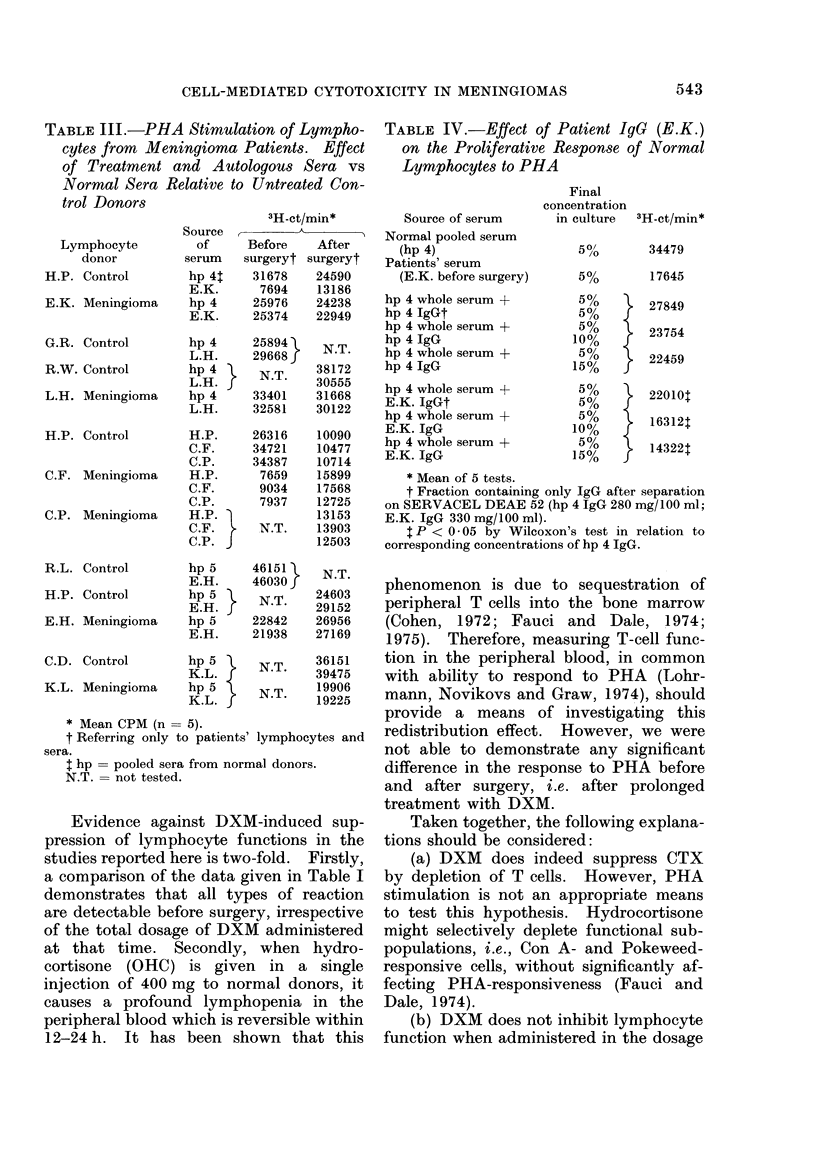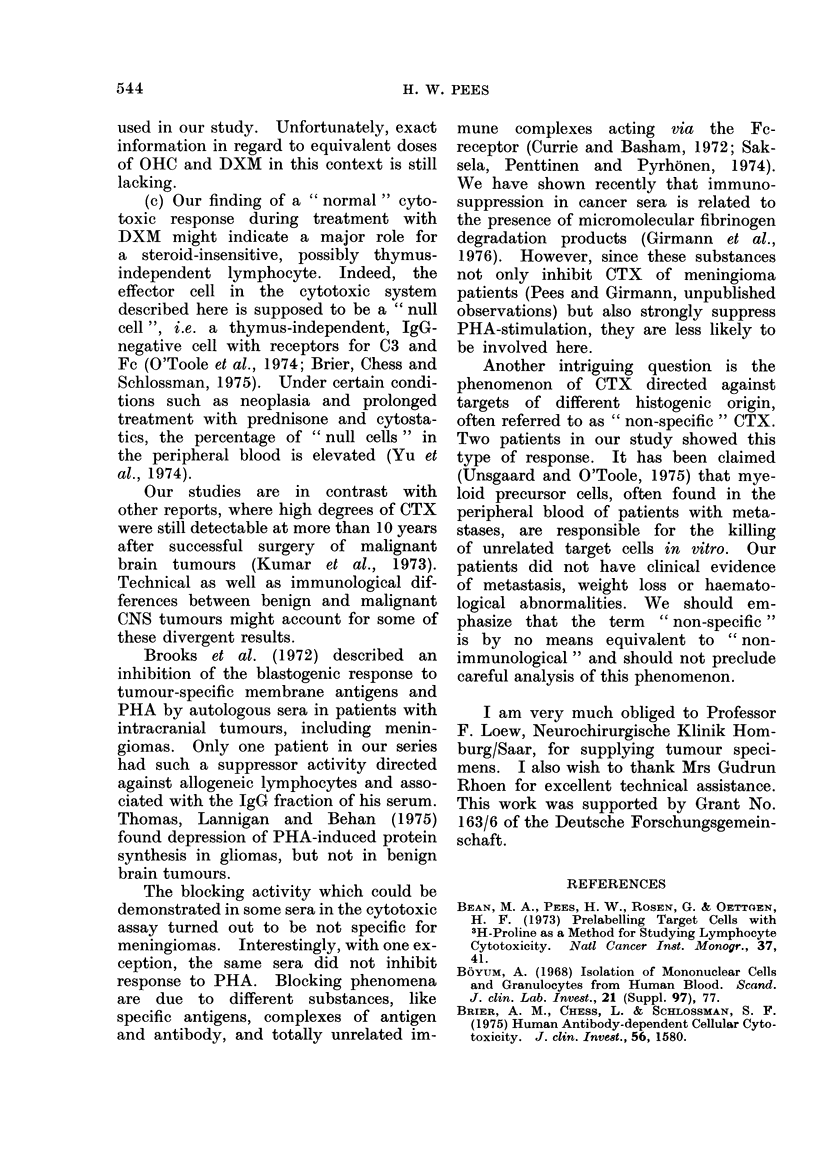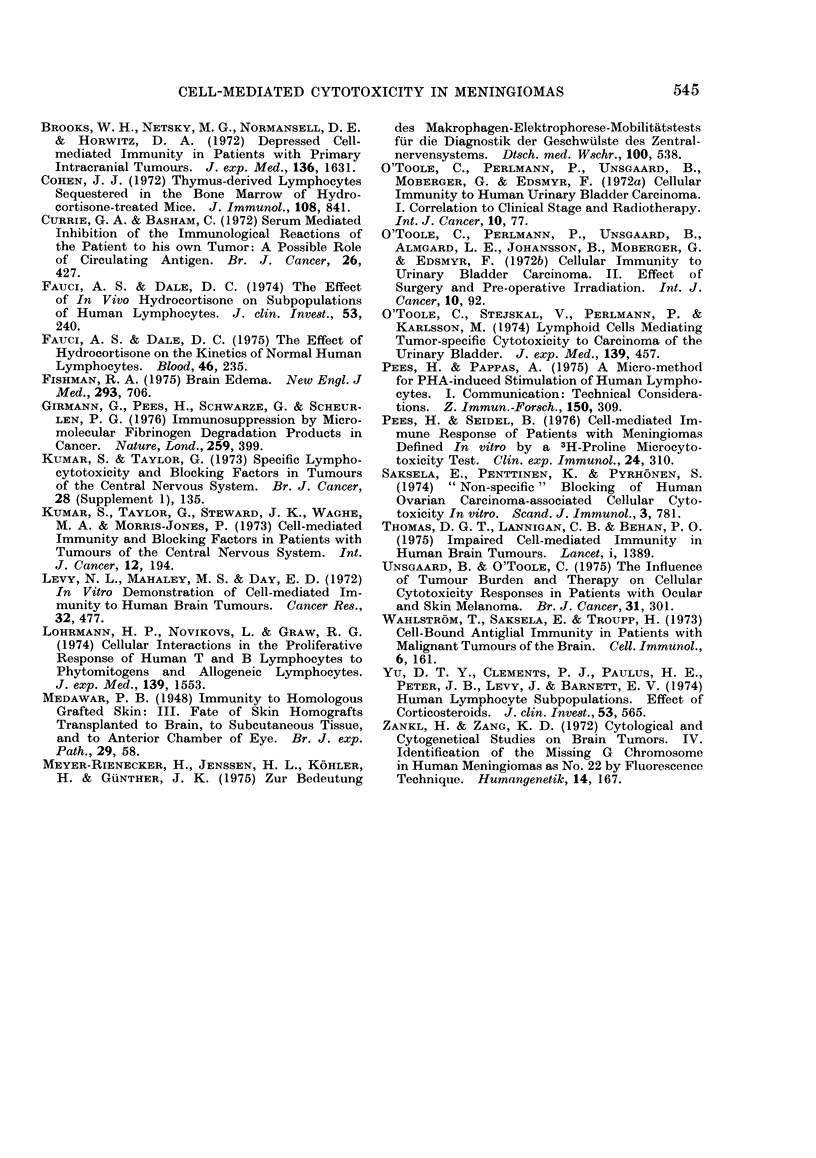# Influence of surgery and dexamethasone on cell-mediated immune responses in patients with meningiomas.

**DOI:** 10.1038/bjc.1977.87

**Published:** 1977-05

**Authors:** H. W. Pees

## Abstract

**Images:**


*Br. J. Cancer* (1977) **35**, 537

### INFLUENCE OF SURGERY AND DEXAMETHASONE ON CELL-MEDIATED IMMUNE RESPONSES IN PATIENTS WITH MENINGIOMAS

H. W. PEES

*From the I. Medizinische Universitätsklinik, Homburg/Saar, West Germany*

Received 6 September 1976 Accepted 12 January 1977

**Summary.—Cell-mediated cytotoxicity (CTX) was studied in meningioma patients before and within 2 weeks of complete excision of the tumour, using the [$^3$H]-proline microcytotoxicity test. Three of 7 patients tested before surgery showed specific CTX, 2 revealed a " non-specific " (tumour-unrelated) response, and 2 were non-reactive. After surgery, CTX decreased from 84 to 50% in one patient and became negative in 2 others previously positive. One of 2 patients showing " non-specific " CTX preoperatively became positive, while the other remained unchanged.**

**All patients were receiving dexamethasone (DXM) at the time they were tested. Lymphocyte responses to PHA were not significantly different before or after surgery (*i.e.* after prolonged treatment with DXM), from healthy controls.**

**Blocking activity could be detected in the sera of all 3 patients before surgery. This activity was not specific for meningiomas. Paradoxically, the same sera did not inhibit the proliferative response to PHA. Serum from only one patient consistently suppressed the blastogenic response of homologous lymphocytes to PHA. Inhibitory activity was associated with the IgG fraction of his serum.**

MANY reports have been published estimating immune responses against human brain tumours *in vitro* (Brooks *et al.*, 1972; Levy, Mahaley and Day, 1972; Kumar and Taylor, 1973; Kumar *et al.*, 1973; Wahlström, Saksela and Troupp, 1973; Meyer-Rienecker *et al.*, 1975). Although these reports demonstrate that the brain can no longer be regarded as an " immunologically privileged site " (Medawar, 1948), results are still conflicting.

In order to eliminate at least some of the technical problems, we used a well-defined [$^3$H]-proline microcytotoxicity test (Bean *et al.*, 1973). A benign tumour (meningioma) was initially selected for study, since such lesions are less heterogenous and easier to grow in tissue culture than malignant tumours of the central nervous system (CNS). Meningiomas are common intracranial tumours and are usually excised completely. Most of these tumours have a definite chromosome loss, both *in vivo* and *in vitro* (Zankl and Zang, 1972).

In a previous study, we demonstrated the occurrence of a specific cytotoxic immune response in about 65% of post-operative meningioma patients tested *in vitro* (Pees and Seidel, 1976). The aim of the present study was to test the effect of surgery on cellular cytotoxicity (CTX). Part of the study also assessed the influence of treatment with dexamethasone (DXM), a drug now widely used in neurosurgery of CNS tumours.

### MATERIALS AND METHODS

*Target cells (T)*.—Biopsy specimens of CNS tumours were carefully dissected, washed several times and trypsinized. Primary

Correspondence: Dr H. W. Pees, I. Medizinische Universitätsklinik, D-6650 Homburg/Saar, West Germany.

cultures were established either from single-cell suspensions or small pieces of tumour. All cultures were maintained in Eagle's minimal essential medium (MEM) with 1% non-essential amino acids (NEAA) supplemented with 2 mM glutamine, streptomycin (100 $\mu$g/ml), penicillin (100 i.u./ml), and 15% foetal calf serum (FCS). All meningioma explants grew rapidly, whereas glioblastoma tissues were much more difficult to establish, either on account of the proliferation of contaminating fibroblasts or the paucity of tumour cells after one or two passages. One line originating from a glioblastoma multiforme (I.F.) appeared satisfactory by morphological criteria and growth characteristics, and was used as a control. Normal fibroblasts were obtained from explants of skin biopsy specimens.

All cultures were routinely checked for fungal and bacterial contamination, and discarded if infected. The cells were frozen in liquid $N_2$ as early as possible, and tested during the first few passages.

*Effector cells* (*E*).—For cytotoxicity experiments, lymphoid cells were purified from venous blood by defibrination, sedimentation with gelatin, and incubation on a nylon wool column for 30 min at 37°C to remove adherent cells. After elution from the column, the cells were treated with 0·84% $NH_4Cl$ and washed ×3 (O'Toole *et al.*, 1972*a*). The final culture medium was MEM with 10% human AB Rh+ serum. Viability was always greater than 95% as judged by trypan blue exclusion.

For determination of the proliferative response to phytohaemagglutinin (PHA), peripheral blood lymphocytes were separated by defibrination and centrifugation over a discontinuous Ficoll–Hypaque gradient (Boyum, 1968). The cells were washed ×3 and final suspensions prepared in MEM containing 10% human AB $Rh^+$ serum.

*Sera.*—Sera were obtained from clotted aseptically drawn venous blood. Red cells were removed by centrifugation at 900 *g* for 10 min. All sera were heat-inactivated at 56°C for 30 min, sterilized by millipore filtration, and stored in aliquots at −20°C until use. In some experiments, several normal sera from AB Rh+ donors were pooled. Each serum was first tested for its ability to support growth of target cells and mitogen-stimulated lymphocytes.

*Microcytotoxicity test.*—Labelling of target cells with [$^3$H]-proline was performed as described elsewhere (Bean *et al.*, 1973). Briefly, monolayers were washed in T30 plastic flasks with MEM not containing NEAA and incubated with 50 $\mu$Ci/ml of [$^3$H]-proline in proline-deficient MEM for 16–18 h (No. TMM 160, sp. act. 20–30 Ci/mmol, Commissariat à l'energie atomique, France). Thereafter the cells were washed with excess amounts of NEAA, trypsinized, and seeded into prefilled (0·1 ml MEM with 10% human AB Rh+ serum) Falcon 3040 microtest plates with a Hamilton syringe delivering 10 $\mu$l containing 1000 viable cells. Target cells were allowed to attach overnight and effector cells added in 0·1 ml containing $2 \times 10^5$ cells (E : T ratio 200 : 1). The plates were incubated for 50–52 h, and then inverted, washed ×2 in PBS with 5% FBS, and processed for residual [$^3$H]-proline as described previously (Pees and Seidel, 1976). Each assay was performed in at least 5 replicates.

Reduction (CTX) of target cell $^3$H (ct/min) by test lymphocytes was compared with control lymphocytes giving the highest ct/min on meningioma tissue according to the formula:

$$\% \text{ CTX} = 100 \times \frac{\begin{array}{c}\text{Highest residual ct/min}\\ \text{with control lymphocytes}\end{array} - \begin{array}{c}\text{Residual ct/min with}\\ \text{test lymphocytes}\end{array}}{\begin{array}{c}\text{Highest residual ct/min}\\ \text{with control lymphocytes}\end{array}}$$

To obtain biologically meaningful results, a minimum of 20% reduction was taken as a positive cytotoxic reaction. Statistical analysis was estimated by Wilcoxon's test with $P < 0{\cdot}05$ representing a significant difference.

Non-specific CTX was defined as a " positive " effect on targets of more than one histogenic origin. Specific CTX denoted a " positive " reaction by an effector cell preparation limited to meningioma targets.

*Lymphocyte culture.*—A volume of 0·1 ml MEM + 10% human AB Rh+ serum containing $2 \times 10^5$ cells was added to a microtest plate (Falcon No. 3040). An additional 0·1 ml of medium containing PHA-P (Difco)

was added to give a final volume of 0·2 ml and 25 μg PHA/ml culture fluid. Cultures were set up in at least 5 replicates, and placed in a 37 °C incubator with a humidified atmosphere and 5% $CO_2$ for 66 h. DNA synthesis was measured by labelling with 1 μCi of [$^3$H] thymidine (sp. act. 400 mCi/mmol) 16 h before harvesting. Cultures were collected by precipitation on to glass-fibre filters with a multiple automatic sample harvester. The optimal conditions for this system using human lymphocytes have been defined elsewhere (Pees and Pappas, 1975).

*Blocking studies.*—Test sera were added at least 60 min before the addition of effector cells. In some experiments, target cells were plated in 10% normal serum and blocking was determined by adding 10 μl of test serum, the control having the same volume of normal serum. Other experiments were performed, using several plates with target cells attached in MEM containing either normal sera or test sera from the outset. In either event, blocking activity was calculated from the formula:

$$\% \text{ Blocking} = \frac{\%\text{ CTX in normal sera} - \%\text{ CTX in autologous sera}}{\%\text{ CTX in normal sera}} \times 100.$$

In PHA-stimulated lymphocyte cultures test sera or purified IgG were added before the addition of PHA.

*Purification of Immunoglobulin G.*—Whole serum was precipitated with 50% ammonium sulphate and the sediment dialysed against 0·01 M phosphate buffer, pH 7·5. IgG was obtained by column separation on DEAE cellulose (Servacel DE 52, Serva, Heidelberg, Germany) previously equilibrated with this buffer. Protein not absorbed under these conditions was dialysed against phosphate-buffered saline, pH 7·3, concentrated to the original serum volume by ultrafiltration, and passed through a millipore filter (0·22 μm). These fractions contained only IgG as judged by immunoelectrophoresis; a quantitative measurement was obtained by radial immunodiffusion.

*Patients.*—All meningioma patients were tested within 1 week before surgery and retested between 7 and 15 days after total excision of their tumour. No attempt was made to eliminate donors with leucocytosis, transfusion history, or complicating infections. All patients with CNS tumours had received DXM for 1–8 days when first tested, the mean dosage being 12–16 mg a day i.v. Details of treatment are given in Table I.

Control donors were healthy volunteers or patients with brain tumours of different histological type including glioblastomas and brain metastases.

### RESULTS

### *Effect of surgery on lymphocyte cytotoxicity (CTX)*

Meningioma explants usually gre was a 2-dimensional reticulum of large, spindle-shaped cells. Sometimes typical psammoma bodies appeared even in later passages (Fig. 1).

Seven meningioma patients could be tested both before and shortly after surgery. Three showed a specific destruction of meningioma cells (27–84% CTX) when tested preoperatively, 2 were negative and another 2 met the criteria of a " non-specific " response. It is noteworthy that in these latter cases an almost total destruction of tumour cells was observed early in the incubation period. This phenomenon did not correlate with transfusion history, recent infection or the composition of effector cells. " Non-specific " CTX on meningioma cells decreased only slightly after surgery, whereas allogeneic fibroblasts were much less affected postoperatively.

As can be seen from Table I and Fig. 2, negative cases remained unchanged, and 2/3 patients positive before surgery became negative. Experiments were also performed using glioblastoma target cells (I.F.) to investigate the specificity of the reaction further. In no case did lymphocytes reacting specifically on meningioma targets show a significant destruction of glioblastoma cells.

### *Influence of treatment with dexamethasone on CTX*

No correlation could be found between

TABLE I.—*Effect of Surgery on Lymphocytotoxicity for Meningioma Cells and Allogeneic Fibroblasts**

| | | | Target cell: Meningioma | | Target cell: Fibroblasts | | |
|---|---|---|---|---|---|---|---|
| Exp. | Lymphocyte donor | Treatment | Residual ct/min† | % Reduction‡ | Residual ct/min | % Reduction | Interpretation§ |
| Ia | Ref. donor I | — | 1993±493 | — | 3184±121 | — | — |
| | Meningioma I | DXM 8 days ‖ | 192±55 | 90¶ | 181±65 | 94¶ | Non-specific |
| Ib | Ref. donor II | — | 2464±199 | — | 2523±89 | — | — |
| | Meningioma I | DXM 15 days, 7 days post excis. | 314±37 | 87¶ | 995±263 | 61¶ | Non-specific |
| IIa | Ref. donor III | — | 1910±110 | — | 5531±338 | — | — |
| | Meningioma II | DXM 1 day | 1183±102 | 38¶ | 5919±137 | 0 | Specific |
| IIb | Ref. donor III | — | 1093±174 | — | 3962±185 | — | — |
| | Meningioma II | DXM 8 days, 6 days post excis. | 1119±186 | 0 | 3994±306 | 0 | Negative |
| IIIa | Ref. donor I | — | 1976±72 | — | 4185±187 | — | — |
| | Meningioma III | DXM 4 days | 1452±239 | 27¶ | 4146±492 | N.S. | Specific |
| IIIb | Ref. donor IV | — | 1963±136 | — | 2297±823 | — | — |
| | Meningioma III | DXM 22 days, 11 days post excis. | 1898±24 | N.S. | 3130±383 | 0 | Negative |
| IVa | Ref. donor V | — | 2269±223 | — | 2141±174 | — | — |
| | Meningioma III | No DXM, 18 days post excis. | 2169±91 | N.S. | 1820±82 | N.S. | Negative |
| | Meningioma IV | DXM 3 days | 2148±192 | N.S. | 1973±186 | N.S. | Negative |
| | Meningioma V | DXM 2 days | 354±161 | 84¶ | 1777±201 | N.S. | Specific |
| IVb | Ref. donor V | — | 644±110 | — | 1592±112 | — | Specific |
| | Meningioma IV | No DXM, 12 days post excis. | 793±85 | 0 | 1605±56 | 0 | Negative |
| | Meningioma V | No DXM, 8 days post excis. | 323±92 | 50¶ | 1495±131 | N.S. | Specific |
| Va | Ref. donor VI | — | 4372±127 | — | 1903±130 | — | — |
| | Meningioma VI | DXM 8 days | 4426±225 | 0 | 1604±281 | N.S. | Negative |
| Vb | Ref. donor II | — | 1293±144 | — | 1516±67 | — | — |
| | Meningioma VI | No DXM, 15 days post excis. | 1245±127 | N.S. | 1566±89 | 0 | Negative |
| VIa | Ref. donor VII | — | 2234±148 | — | 4435±746 | — | — |
| | Meningioma VII | DXM 2 days | 129±31 | 94¶ | 2451±475 | 45¶ | Non-specific |
| VIb | Ref. donor VIII | — | 2451±275 | — | 1238±93 | — | — |
| | Meningioma VII | No DXM, 8 days post excis. | 573±77 | 77¶ | 1244±83 | 0 | Specific |

* Data of reference donors and meningioma patients only.
† Mean $^3$H-ct/min ± s.d. (n = 6) after 50 h of incubation. Ratio effector cells : target cells 200 : 1.
‡ Relative to reference donor.
§ Specific = at least 20% reduction only on meningioma cells. Non-specific = at least 20% reduction on meningioma cells and fibroblasts.
‖ Duration of treatment with dexamethasone (DXM see text).
¶ Significant at $P < 0{\cdot}05$ by Wilcoxon's test.
N.S. Not significant.

preoperative CTX and duration of therapy with DXM. Likewise, postoperative responses did not show any relation to the total dosage of steroids given at that time. When treatment ceased, no increase of CTX could be observed in previously positive (Nos. III, V) or negative patients (Nos. IV, VI).

*Blocking experiments*

Autologous serum collected before surgery inhibited CTX: an "arming" effect was not observed (Table II). In one patient (C.F.) showing a specific response even after surgery, the postoperative serum had decreased blocking activity. In order to test the specificity

FIG. 1.—Meningioma J.S., passage 2; phase contrast. ×95.

FIG. 2.—Cytotoxicity of meningioma patients before and within 2 weeks after surgery. Open circles, " non-specific " CTX.

of this inhibitory effect, lymphocytes from patient K.L. were incubated on allogeneic fibroblasts and meningioma cells using both normal and autologous serum. Since in normal serum destruction of all targets occurred and autologous serum was able to suppress this effect, the conclusion seems to be justified that this blocking activity was "non-specific".

### *Relation to blood group substances*

Agglutination of contaminating red blood cells in the effector cell preparations by certain test sera was observed, as expected from blood group differences. A cytotoxic effect, however, did not occur even in those combinations where test sera contained isoantibodies directed against blood group antigens of the target cell donor (ABO system). No attempt was made to test the expression of these antigens on the target cells in question.

TABLE II.—*Inhibition of Lymphocytotoxicity by Sera from Meningioma Patients before and after Surgery*

| Exp.* | Immune lymphocyte donor (meningioma) | Treatment† | Target cell | % Reduction relative to reference donor after incubation with: Control serum | Patient's serum | % Blocking‡ |
|---|---|---|---|---|---|---|
| IIa | E.K. | Before | Meningioma (R.M.) | 38 | 27 | 29 |
| IIb | | After | Meningioma (R.M.) | 0 | 0 | — |
| | | After | Meningioma (E.K.) | 6 N.S. | 0 | — |
| IIIa | L.H. | Before | Meningioma (J.S.) | 27 | 16 | 41 |
| IIIb | | After | Meningioma (J.S.) | 3 N.S. | 6 N.S. | — |
| IVa | C.F. | Before | Meningioma (J.S.) | 84 | 50 | 40 |
| IVb | | After | Meningioma (C.F.) | 50 | 40 | 20 |
| Va | E.H. | Before | Meningioma (J.S.) | 0 | 12 N.S. | — |
| Vb | | After | Meningioma (J.S.) | N.S. | 0 | — |
| VIa | K.L. | Before | Meningioma (M.Z.) | 94 | 9 N.S. | 90 |
| | | Before | Skin fibroblasts (A.N.) | 45 | 4 N.S. | 91 |
| VIb | | After | Meningioma (K.L.) | 77 | 10 N.S. | 87 |

* See Table I.

† Date of drawing blood for both lymphocytes and patients' sera relative to surgery.

‡ $\% \text{ Blocking} = \frac{\% \text{ CTX in normal serum} - \% \text{ CTX in autologous serum}}{\% \text{ CTX in normal serum}} \times 100.$

N.S. Not significant.

*Lymphocyte culture*

Table III summarizes the results of PHA stimulation in 5 meningioma patients compared to normal donors. In no case could a decrease of DNA synthesis be seen after prolonged steroid administration. On the contrary, in 2 patients, an increase of thymidine incorporation was observed postoperatively. No significant difference could be demonstrated in autologous sera before and after surgery. One patient (E.K.) had a reproducible inhibitory activity in his serum when tested on allogeneic normal lymphocytes. Separation procedures revealed that this effect was associated with a fraction containing only IgG (Table IV). There was no history of multiple pregnancies or transfusions in this donor.

### DISCUSSION

Previous studies of other authors (O'Toole *et al.*, 1972*b*) and from our own laboratory (Pees and Seidel, 1976) have suggested that cell-mediated immunity as measured *in vitro* by a microcytotoxicity assay might depend on the presence of a critical tumour mass in the body. In meningioma patients the highest response rate was seen during the first 3 weeks. The observation of a rapidly disappearing CTX in one patient tested on several occasions postoperatively prompted us to test the effect of surgery on CTX in these tumours.

Our results demonstrate a marked decrease or even disappearance of CTX following surgery. In all patients the tumour had been removed completely. However, since there is now widespread conviction that dexamethasone may dramatically reduce peri-tumoral oedema (Fishman, 1975), a considerable number of patients with CNS tumours will be treated with this drug for a certain period before and after surgery. Thus, most of our patients were receiving treatment already when they were admitted to the department of neurosurgery. We therefore decided to test all patients during therapy, though we were aware that the immunological situation would be even more complex.

TABLE III.—*PHA Stimulation of Lymphocytes from Meningioma Patients. Effect of Treatment and Autologous Sera vs Normal Sera Relative to Untreated Control Donors*

| Lymphocyte donor | Source of serum | $^3$H-ct/min* Before surgery† | After surgery† |
|---|---|---|---|
| H.P. Control | hp 4‡ | 31678 | 24590 |
| | E.K. | 7694 | 13186 |
| E.K. Meningioma | hp 4 | 25976 | 24238 |
| | E.K. | 25374 | 22949 |
| G.R. Control | hp 4 | 25894 | N.T. |
| | L.H. | 29668 | |
| R.W. Control | hp 4 | N.T. | 38172 |
| | L.H. | | 30555 |
| L.H. Meningioma | hp 4 | 33401 | 31668 |
| | L.H. | 32581 | 30122 |
| H.P. Control | H.P. | 26316 | 10090 |
| | C.F. | 34721 | 10477 |
| | C.P. | 34387 | 10714 |
| C.F. Meningioma | H.P. | 7659 | 15899 |
| | C.F. | 9034 | 17568 |
| | C.P. | 7937 | 12725 |
| C.P. Meningioma | H.P. | N.T. | 13153 |
| | C.F. | | 13903 |
| | C.P. | | 12503 |
| R.L. Control | hp 5 | 46151 | N.T. |
| | E.H. | 46030 | |
| H.P. Control | hp 5 | N.T. | 24603 |
| | E.H. | | 29152 |
| E.H. Meningioma | hp 5 | 22842 | 26956 |
| | E.H. | 21938 | 27169 |
| C.D. Control | hp 5 | N.T. | 36151 |
| | K.L. | | 39475 |
| K.L. Meningioma | hp 5 | N.T. | 19906 |
| | K.L. | | 19225 |

* Mean CPM (n = 5).

† Referring only to patients' lymphocytes and sera.

‡ hp = pooled sera from normal donors.

N.T. = not tested.

TABLE IV.—*Effect of Patient IgG (E.K.) on the Proliferative Response of Normal Lymphocytes to PHA*

| Source of serum | Final concentration in culture | $^3$H-ct/min* |
|---|---|---|
| Normal pooled serum (hp 4) | 5% | 34479 |
| Patients' serum (E.K. before surgery) | 5% | 17645 |
| hp 4 whole serum + | 5% | 27849 |
| hp 4 IgG† | 5% | |
| hp 4 whole serum + | 5% | 23754 |
| hp 4 IgG | 10% | |
| hp 4 whole serum + | 5% | 22459 |
| hp 4 IgG | 15% | |
| hp 4 whole serum + | 5% | 22010‡ |
| E.K. IgG† | 5% | |
| hp 4 whole serum + | 5% | 16312‡ |
| E.K. IgG | 10% | |
| hp 4 whole serum + | 5% | 14322‡ |
| E.K. IgG | 15% | |

* Mean of 5 tests.
† Fraction containing only IgG after separation on SERVACEL DEAE 52 (hp 4 IgG 280 mg/100 ml; E.K. IgG 330 mg/100 ml).
‡ $P < 0 \cdot 05$ by Wilcoxon's test in relation to corresponding concentrations of hp 4 IgG.

Evidence against DXM-induced suppression of lymphocyte functions in the studies reported here is two-fold. Firstly, a comparison of the data given in Table I demonstrates that all types of reaction are detectable before surgery, irrespective of the total dosage of DXM administered at that time. Secondly, when hydrocortisone (OHC) is given in a single injection of 400 mg to normal donors, it causes a profound lymphopenia in the peripheral blood which is reversible within 12–24 h. It has been shown that this phenomenon is due to sequestration of peripheral T cells into the bone marrow (Cohen, 1972; Fauci and Dale, 1974; 1975). Therefore, measuring T-cell function in the peripheral blood, in common with ability to respond to PHA (Lohrmann, Novikovs and Graw, 1974), should provide a means of investigating this redistribution effect. However, we were not able to demonstrate any significant difference in the response to PHA before and after surgery, *i.e.* after prolonged treatment with DXM.

Taken together, the following explanations should be considered:

(a) DXM does indeed suppress CTX by depletion of T cells. However, PHA stimulation is not an appropriate means to test this hypothesis. Hydrocortisone might selectively deplete functional subpopulations, *i.e.*, Con A- and Pokeweed-responsive cells, without significantly affecting PHA-responsiveness (Fauci and Dale, 1974).

(b) DXM does not inhibit lymphocyte function when administered in the dosage used in our study. Unfortunately, exact information in regard to equivalent doses of OHC and DXM in this context is still lacking.

(c) Our finding of a " normal " cytotoxic response during treatment with DXM might indicate a major role for a steroid-insensitive, possibly thymus-independent lymphocyte. Indeed, the effector cell in the cytotoxic system described here is supposed to be a " null cell ", *i.e.* a thymus-independent, IgG-negative cell with receptors for C3 and Fc (O'Toole *et al.*, 1974; Brier, Chess and Schlossman, 1975). Under certain conditions such as neoplasia and prolonged treatment with prednisone and cytostatics, the percentage of " null cells " in the peripheral blood is elevated (Yu *et al.*, 1974).

Our studies are in contrast with other reports, where high degrees of CTX were still detectable at more than 10 years after successful surgery of malignant brain tumours (Kumar *et al.*, 1973). Technical as well as immunological differences between benign and malignant CNS tumours might account for some of these divergent results.

Brooks *et al.* (1972) described an inhibition of the blastogenic response to tumour-specific membrane antigens and PHA by autologous sera in patients with intracranial tumours, including meningiomas. Only one patient in our series had such a suppressor activity directed against allogeneic lymphocytes and associated with the IgG fraction of his serum. Thomas, Lannigan and Behan (1975) found depression of PHA-induced protein synthesis in gliomas, but not in benign brain tumours.

The blocking activity which could be demonstrated in some sera in the cytotoxic assay turned out to be not specific for meningiomas. Interestingly, with one exception, the same sera did not inhibit response to PHA. Blocking phenomena are due to different substances, like specific antigens, complexes of antigen and antibody, and totally unrelated immune complexes acting *via* the Fc-receptor (Currie and Basham, 1972; Saksela, Penttinen and Pyrhönen, 1974). We have shown recently that immunosuppression in cancer sera is related to the presence of micromolecular fibrinogen degradation products (Girmann *et al.*, 1976). However, since these substances not only inhibit CTX of meningioma patients (Pees and Girmann, unpublished observations) but also strongly suppress PHA-stimulation, they are less likely to be involved here.

Another intriguing question is the phenomenon of CTX directed against targets of different histogenic origin, often referred to as " non-specific " CTX. Two patients in our study showed this type of response. It has been claimed (Unsgaard and O'Toole, 1975) that myeloid precursor cells, often found in the peripheral blood of patients with metastases, are responsible for the killing of unrelated target cells *in vitro*. Our patients did not have clinical evidence of metastasis, weight loss or haematological abnormalities. We should emphasize that the term " non-specific " is by no means equivalent to " non-immunological " and should not preclude careful analysis of this phenomenon.

I am very much obliged to Professor F. Loew, Neurochirurgische Klinik Homburg/Saar, for supplying tumour specimens. I also wish to thank Mrs Gudrun Rhoen for excellent technical assistance. This work was supported by Grant No. 163/6 of the Deutsche Forschungsgemeinschaft.

### REFERENCES

BEAN, M. A., PEES, H. W., ROSEN, G. & OETTGEN, H. F. (1973) Prelabelling Target Cells with $^{3}$H-Proline as a Method for Studying Lymphocyte Cytotoxicity. *Natl Cancer Inst. Monogr.*, **37**, 41.

BÖYUM, A. (1968) Isolation of Mononuclear Cells and Granulocytes from Human Blood. *Scand. J. clin. Lab. Invest.*, **21** (Suppl. **97**), 77.

BRIER, A. M., CHESS, L. & SCHLOSSMAN, S. F. (1975) Human Antibody-dependent Cellular Cytotoxicity. *J. clin. Invest.*, **56**, 1580.

BROOKS, W. H., NETSKY, M. G., NORMANSELL, D. E. & HORWITZ, D. A. (1972) Depressed Cell-mediated Immunity in Patients with Primary Intracranial Tumours. *J. exp. Med.*, **136**, 1631.

COHEN, J. J. (1972) Thymus-derived Lymphocytes Sequestered in the Bone Marrow of Hydrocortisone-treated Mice. *J. Immunol.*, **108**, 841.

CURRIE, G. A. & BASHAM, C. (1972) Serum Mediated Inhibition of the Immunological Reactions of the Patient to his own Tumor: A Possible Role of Circulating Antigen. *Br. J. Cancer*, **26**, 427.

FAUCI, A. S. & DALE, D. C. (1974) The Effect of *In Vivo* Hydrocortisone on Subpopulations of Human Lymphocytes. *J. clin. Invest.*, **53**, 240.

FAUCI, A. S. & DALE, D. C. (1975) The Effect of Hydrocortisone on the Kinetics of Normal Human Lymphocytes. *Blood*, **46**, 235.

FISHMAN, R. A. (1975) Brain Edema. *New Engl. J Med.*, **293**, 706.

GIRMANN, G., PEES, H., SCHWARZE, G. & SCHEURLEN, P. G. (1976) Immunosuppression by Micromolecular Fibrinogen Degradation Products in Cancer. *Nature, Lond.*, **259**, 399.

KUMAR, S. & TAYLOR, G. (1973) Specific Lymphocytotoxicity and Blocking Factors in Tumours of the Central Nervous System. *Br. J. Cancer*, **28** (Supplement 1), 135.

KUMAR, S., TAYLOR, G., STEWARD, J. K., WAGHE, M. A. & MORRIS-JONES, P. (1973) Cell-mediated Immunity and Blocking Factors in Patients with Tumours of the Central Nervous System. *Int. J. Cancer*, **12**, 194.

LEVY, N. L., MAHALEY, M. S. & DAY, E. D. (1972) *In Vitro* Demonstration of Cell-mediated Immunity to Human Brain Tumours. *Cancer Res.*, **32**, 477.

LOHRMANN, H. P., NOVIKOVS, L. & GRAW, R. G. (1974) Cellular Interactions in the Proliferative Response of Human T and B Lymphocytes to Phytomitogens and Allogeneic Lymphocytes. *J. exp. Med.*, **139**, 1553.

MEDAWAR, P. B. (1948) Immunity to Homologous Grafted Skin: III. Fate of Skin Homografts Transplanted to Brain, to Subcutaneous Tissue, and to Anterior Chamber of Eye. *Br. J. exp. Path.*, **29**, 58.

MEYER-RIENECKER, H., JENSSEN, H. L., KÖHLER, H. & GÜNTHER, J. K. (1975) Zur Bedeutung des Makrophagen-Elektrophorese-Mobilitätstests für die Diagnostik der Geschwülste des Zentralnervensystems. *Dtsch. med. Wschr.*, **100**, 538.

O'TOOLE, C., PERLMANN, P., UNSGAARD, B., MOBERGER, G. & EDSMYR, F. (1972*a*) Cellular Immunity to Human Urinary Bladder Carcinoma. I. Correlation to Clinical Stage and Radiotherapy. *Int. J. Cancer*, **10**, 77.

O'TOOLE, C., PERLMANN, P., UNSGAARD, B., ALMGARD, L. E., JOHANSSON, B., MOBERGER, G. & EDSMYR, F. (1972*b*) Cellular Immunity to Urinary Bladder Carcinoma. II. Effect of Surgery and Pre-operative Irradiation. *Int. J. Cancer*, **10**, 92.

O'TOOLE, C., STEJSKAL, V., PERLMANN, P. & KARLSSON, M. (1974) Lymphoid Cells Mediating Tumor-specific Cytotoxicity to Carcinoma of the Urinary Bladder. *J. exp. Med.*, **139**, 457.

PEES, H. & PAPPAS, A. (1975) A Micro-method for PHA-induced Stimulation of Human Lymphocytes. I. Communication: Technical Considerations. *Z. Immun.-Forsch.*, **150**, 309.

PEES, H. & SEIDEL, B. (1976) Cell-mediated Immune Response of Patients with Meningiomas Defined *In vitro* by a $^3$H-Proline Microcytotoxicity Test. *Clin. exp. Immunol.*, **24**, 310.

SAKSELA, E., PENTTINEN, K. & PYRHÖNEN, S. (1974) "Non-specific" Blocking of Human Ovarian Carcinoma-associated Cellular Cytotoxicity *In vitro*. *Scand. J. Immunol.*, **3**, 781.

THOMAS, D. G. T., LANNIGAN, C. B. & BEHAN, P. O. (1975) Impaired Cell-mediated Immunity in Human Brain Tumours. *Lancet*, i, 1389.

UNSGAARD, B. & O'TOOLE, C. (1975) The Influence of Tumour Burden and Therapy on Cellular Cytotoxicity Responses in Patients with Ocular and Skin Melanoma. *Br. J. Cancer*, **31**, 301.

WAHLSTRÖM, T., SAKSELA, E. & TROUPP, H. (1973) Cell-Bound Antiglial Immunity in Patients with Malignant Tumours of the Brain. *Cell. Immunol.*, **6**, 161.

YU, D. T. Y., CLEMENTS, P. J., PAULUS, H. E., PETER, J. B., LEVY, J. & BARNETT, E. V. (1974) Human Lymphocyte Subpopulations. Effect of Corticosteroids. *J. clin. Invest.*, **53**, 565.

ZANKL, H. & ZANG, K. D. (1972) Cytological and Cytogenetical Studies on Brain Tumors. IV. Identification of the Missing G Chromosome in Human Meningiomas as No. 22 by Fluorescence Technique. *Humangenetik*, **14**, 167.